# Transcriptome profiling of the rumen epithelium of beef cattle differing in residual feed intake

**DOI:** 10.1186/s12864-016-2935-4

**Published:** 2016-08-09

**Authors:** Rebecca S. G. Kong, Guanxiang Liang, Yanhong Chen, Paul Stothard, Le Luo Guan

**Affiliations:** Department of Agricultural, Food and Nutritional Science, Agriculture/Forestry Centre, University of Alberta, 416F, Edmonton, AB T6G 2P5 Canada

**Keywords:** Feed efficiency, Residual feed intake, RNA-sequencing, Beef cattle, Rumen epithelium, Transcriptome

## Abstract

**Background:**

Feed efficient cattle consume less feed and produce less environmental waste than inefficient cattle. Many factors are known to contribute to differences in feed efficiency, however the underlying molecular mechanisms are largely unknown. Our study aimed to understand how host gene expression in the rumen epithelium contributes to differences in residual feed intake (RFI), a measure of feed efficiency, using a transcriptome profiling based approach.

**Results:**

The rumen epithelial transcriptome from highly efficient (low (L-) RFI, *n* = 9) and inefficient (high (H-) RFI, *n* = 9) Hereford x Angus steers was obtained using RNA-sequencing. There were 122 genes differentially expressed between the rumen epithelial tissues of L- and H- RFI steers (*p* < 0.05) with 85 up-regulated and 37 down-regulated in L-RFI steers. Functional analysis of up-regulated genes revealed their involvement in acetylation, remodeling of adherens junctions, cytoskeletal dynamics, cell migration, and cell turnover. Additionally, a weighted gene co-expression network analysis (WGCNA) identified a significant gene module containing 764 genes that was negatively correlated with RFI (*r* = −0.5, *p* = 0.03). Functional analysis revealed significant enrichment of genes involved in modulation of intercellular adhesion through adherens junctions, protein and cell turnover, and cytoskeletal organization that suggest possible increased tissue morphogenesis in the L-RFI steers. Additionally, the L-RFI epithelium had increased expression of genes involved with the mitochondrion, acetylation, and energy generating pathways such as glycolysis, tricarboxylic acid cycle, and oxidative phosphorylation. Further qPCR analysis of steers with different RFI (L-RFI, *n* = 35; M-RFI, *n* = 34; H-RFI, *n* = 35) revealed that the relative mitochondrial genome copy number per cell of the epithelium was positively correlated with RFI (*r* = 0.21, *p* = 0.03).

**Conclusions:**

Our results suggest that the rumen epithelium of L-RFI (efficient) steers may have increased tissue morphogenesis that possibly increases paracellular permeability for the absorption of nutrients and increased energy production to support the energetic demands of increased tissue morphogenesis compared to those of H-RFI (inefficient) animals. Greater expression of mitochondrial genes and lower relative mitochondrial genome copy numbers suggest a greater rate of transcription in the rumen epithelial mitochondria of L-RFI steers. Understanding how host gene expression profiles are associated with RFI could potentially lead to identification of mechanisms behind this trait, which are vital to develop strategies for the improvement of cattle feed efficiency.

**Electronic supplementary material:**

The online version of this article (doi:10.1186/s12864-016-2935-4) contains supplementary material, which is available to authorized users.

## Background

Feed is one of the major costs in beef cattle production, accounting for approximately 70 % of total expenditures [1]. As feed price continues to rise due to factors such as human population growth and decreasing amount of arable land [2], beef cattle feed efficiency has become an increasingly important contributor to reduce the cost of production. Recent efforts in the selection of feed efficient cattle have been focused on residual feed intake (RFI), a measure of feed efficiency that is independent of growth and body weight [3]. The RFI of an individual is calculated as the difference between actual feed intake and expected feed intake based on maintenance and growth requirements [4]. Animals with low (negative) RFI (L-RFI) are considered feed efficient because they consume less feed than expected whereas animals with high (positive) RFI (H-RFI) are feed inefficient due to consuming more feed than expected [4]. Selection for L-RFI animals would not only reduce cost of production, but also decrease the environmental footprint because they produce 28 and 15 % less methane [5] and manure [6], respectively, than H-RFI animals.

Variation in RFI may be due to differences in many biological processes that are influenced by genetic and environmental factors. Physiological processes that have been found to contribute to variation in RFI include those associated with feed intake, digestion, metabolism, activity, and stress [7, 8]. Genetic selection and improvement of RFI is possible since RFI is a moderately heritable trait (0.28 to 0.45) [3, 9, 10], however, the molecular mechanisms underlying RFI are largely unknown. Because variation in RFI is due to differences in many processes, multiple genes from various pathways are likely involved [11–13]. Discovering differences at the molecular level may potentially lead to identification of biomarkers for use in the selection of L-RFI (feed efficient) animals.

In ruminant animals, such as cattle, the rumen may play an important role in feed efficiency. It is the main site of microbial feed digestion and fermentation that produces short-chain fatty acids (SCFAs), which are a major energy source for cattle accounting for up to 80 % of total energy requirements [14, 15]. SCFAs are mainly absorbed from the rumen [16] and metabolized within the rumen epithelium, liver and peripheral tissues to generate energy for use in maintenance, growth and activity [17, 18]. Studies have indicated that SCFAs are absorbed by both simple diffusion [16] and protein-mediated transport through bicarbonate - dependent and - independent mechanisms [19]. Moreover, ketogenic enzymes within the mitochondria of the rumen epithelium play a crucial role in the metabolism of SCFAs, to produce ketone bodies that are an important circulating source of energy throughout the body [20, 21].

Differences in the ability of the rumen epithelium to absorb and metabolize SCFAs may be associated with variation in feed efficiency. Therefore, the objective of this study was to investigate differences in gene expression between the rumen epithelial tissues of feed efficient and inefficient beef cattle using RNA-sequencing (RNA-seq) technology and bioinformatics. We hypothesized that the rumen epithelium of L-RFI (efficient) animals would have greater expression of genes involved in absorption and metabolism of energetic substrates such as SCFAs.

## Methods

### Animals and rumen tissue collection

Hereford x Aberdeen Angus hybrid steers (*n* = 175) were born in the spring of 2006 and raised at the University of Alberta Roy Berg Kinsella Research Station (Alberta, Canada) in accordance with the Canadian Council of Animal Care guidelines [22]. The University of Alberta Animal Care and Use Committee for Livestock approved the experimental protocols (Moore-55-2006).

Steers were under feedlot conditions while feed intake data was collected using the GrowSafe automated feeding system (GrowSafe Systems Ltd., Airdrie, Alberta, Canada). The finishing diet was fed for 90 days and was composed of 56.7 % barley, 28.3 % oats, 10 % alfalfa pellets, and 5 % feedlot supplement (32 % crude protein beef supplement containing Rumensin (400 mg/kg) and 1.5 % canola oil). After slaughter, a 4-cm^2^ piece of rumen tissue was obtained from the central region of the ventral sac and rinsed with sterilized PBS buffer (pH = 6.8) before being placed in a 50 mL tube containing RNAlater solution (Invitrogen, Carlsbad, CA). The samples were then stored at −80 °C until further processing.

RFI values of all steers were calculated as described by Nkrumah et al. (2006) [5] and classified as L-RFI (feed efficient; RFI < −0.5), medium RFI (M-RFI; −0.5 ≤ RFI ≤ 0.5), or H-RFI (feed inefficient; RFI > 0.5), which have been reported by Durunna et al. 2011 [23]. The rumen epithelial tissues were collected from all animals when they were slaughtered and the rumen epithelia from the most extreme L-RFI (*n* = 9; RFI = −1.4 to −2.33 kg/day) and H-RFI (*n* = 9; RFI = 1.32 to 3.23 kg/day) steers (Additional file 1) were selected for transcriptome analysis using RNA-seq.

### RNA extraction and sequencing

After thawing, rumen tissue was placed on a petri dish and papillae (~80 mg) were obtained using sterile scissors and scalpels. The total RNA was extracted from the papillae using mirVana kit (Ambion, Austin, TX) according to the manufacturer’s instructions. RNA integrity of the total RNA samples was assessed using the Agilent 2100 Bioanalyzer (Agilent Technologies, Santa Clara, CA) and Nanodrop 2000c spectrophotometer (Thermo Scientific, Wilmington, DE) was used to measure the concentration and purity. Only samples with RNA integrity greater than 7 were subjected to RNA-seq library construction.

For each of the 18 samples, a cDNA library was generated from 100 ng of total RNA following the protocol of the TruSeq RNA Sample Preparation v2 kit (Illumina, San Diego, CA). First, total RNA was fragmented and cDNA was synthesized through reverse transcription. The resulting double-stranded cDNA was then subjected to end repair and 3’-end adenylation before ligation of index adapters. Libraries were amplified by 15 cycles of PCR, then validated with the Agilent 2200 TapeStation (Agilent Technologies) and quantified with a Qubit fluorometer using a Qubit dsDNA HS Assay Kit (Invitrogen, Carlsbad, CA). The individual indexed libraries were then pooled and sequenced in five lanes on the Illumina HiSeq 2000 system at the McGill University and Genome Quebec Innovation Centre (Quebec, Canada) to obtain high-quality, 100-bp paired-end reads (Average Phred quality score ≥ 33).

### Transcriptome mapping

We used the software package TopHat2 (v2.0.9) [24] with Bowtie2 (v2.1.0) [25] to align the high-quality RNA-seq reads to the bovine reference genome, UMD3.1 (Ensembl v83.31). Samtools (v1.1) [26] was used to sort the BAM alignment files that were generated from TopHat2 by name and these sorted BAM files were converted to SAM files for input to HTSeq-count (v0.6.1) [27], which is a software tool that counts the number of reads per gene using the Ensembl (v83.31) bovine gene annotation.

### Differential gene expression and principal component analysis

Differential gene expression analysis was performed using the Bioconductor (v3.0) [28] package DESeq2 (v1.6.2) [29] in the R statistical software program (v3.1.2) [30]. Firstly, DESeq2 normalized gene count data using the relative log expression method based on “size factors” to account for RNA-seq library size differences and dispersion estimates were calculated. Then, pairwise comparison of expression was made between the L-RFI and H-RFI group for every gene based on a negative binomial model. Fold changes were obtained along with their associated p-values. The Benjamini Hochberg method (B-H) was used to control the false discovery rate (FDR) by adjusting p-values to correct for multiple testing. A gene was defined as differentially expressed (DE) between the L-RFI and H-RFI group if it had a B-H adjusted p-value (FDR) less than 0.05.

DESeq2 was also used for Principal Component Analysis (PCA) to cluster samples based on gene expression data. Firstly, count data was normalized based on library size and regularized log transformed. The top 500 genes with the most variance (i.e. Largest standard deviations) were compared between samples to calculate principal components in order to view clustering in two dimensions.

### Weighted Gene Co-expression Network Analysis (WGCNA)

The WGCNA R software package (v1.41.1) [31] was used to identify modules containing genes that are co-expressed and correlated with the RFI trait. Gene expression data was firstly filtered by keeping genes with at least one read in 50 % of the samples, then normalized by count per million reads (CPM) and Log2 transformed (Log2(CPM + 1)). Removal of the very lowly expressed genes resulted in 14,916 genes for input to WGCNA. Unsigned, weighted correlation network construction and module detection was performed using the automatic one-step function, blockwiseModules. The resulting gene modules were assigned color names by the software and Module-Trait relationships were calculated by Pearson correlation. Modules with statistically significant (*p* < 0.05) correlations were selected for downstream analysis as potential biologically interesting modules associated with the RFI trait. Additionally, a hierarchical clustering dendrogram of samples and trait heatmap was created using unfiltered, Log2CPM data. Sample clustering was based on Euclidean distance and the traits used in the heatmap were residual feed intake (RFI), dry matter intake (DMI), metabolizable energy intake (MEI), birth weight (BirthWT), weaning weight (WeanWT), metabolic mid-weight (MWT), end weight (EndWT), carcass weight (CWT), and average daily gain (ADG).

### Functional gene annotation

Ensembl gene ID lists were converted to gene names and symbols using the Database for Annotation, Visualization, and Integrated Discovery (DAVID) [32, 33] and the R/Bioconductor package, biomaRt (v2.22.0) [34]. DAVID and Ingenuity Pathway Analysis (IPA; QIAGEN Redwood City, www.qiagen.com/ingenuity) were used for functional annotation of DE genes and WGCNA gene modules. In DAVID, we examined genes for enrichment in functional categories such as SP_PIR_Keywords and Kyoto Encyclopedia of Genes and Genomes (KEGG) pathways [35, 36]. To correct for multiple testing in DAVID, p-values were adjusted using B-H correction. Biological terms were considered significant if the adjusted p-value was less than 0.05. The genes input to IPA were compared to the manually curated Ingenuity knowledge base to find significantly enriched canonical pathways, and cellular and molecular processes. Statistical significance (*p* < 0.05) was calculated using the right-tailed Fischer’s Exact Test and B-H corrected for multiple testing.

The identified core transcripts (i.e. genes with at least one read in every sample) were input to the Protein Annotation Through Evolutionary Relationship (PANTHER) [37] gene list analysis tool to determine the core functions of the rumen epithelium. Functional classification of genes in categories such as molecular function and biological process was based on Gene Ontology (GO) annotations.

### Quantitative real-time PCR (qPCR) validation of selected differentially expressed genes

To validate the identified DE genes from RNA-seq, total RNA was extracted from rumen papillae of 30 L-RFI samples (RFI = −0.51 to −2.33 kg/day) and 27 H-RFI samples (RFI = 0.66 to 3.23 kg/day), respectively. Primers targeting five selected DE genes including Trans-2,3-Enoyl-CoA Reductase (*TECR*), Cytochrome C Oxidase Subunit VIIIA (*COX8A*), Solute Carrier Family 25 Member 39 (*SLC25A39*), Pyruvate Kinase M2 (*PKM2*) and Triosephosphate Isomerase 1 (*TPI1*) were designed using Primer-BLAST (NCBI, Bethesda, MD), Primer Express software (Applied Biosystems, Foster City, CA) or obtained through literatures. The specificity of primers was checked with BLAST (NCBI) and the UCSC In-Silico PCR program [38]. Table 1 lists the housekeeping gene (endogenous control) and the five DE genes selected for qPCR validation along with their GenBank accession number, primer sequences, product length and functional pathway.

**Table 1:** Gene name, accession number, functional pathway, primer sequences, and product length of qPCR validated genes

Gene name (symbol)	GenBank accession number	Functional pathway	Forward and reverse primer sequence (5'-3')	Product length (bp)	Reference
Triosephosphate Isomerase 1 (*TPI1*)	NM_001013589	Glycolysis, Gluconeogenesis, Acetylation	Fwd: GGGAGGAAGAACAATCTGGGG	107	This study
			Rev: GCGAAGTCAATGTAGGCGGT		
Trans-2,3-Enoyl-CoA Reductase (*TECR*)	NM_001034748	Biosynthesis of unsaturated fatty acids, Acetylation	Fwd: CCAAGGGCAAGTCCCTGAAG	82	This study
			Rev: AGGTCCCGGAAGTAGAGTGT		
Cytochrome C Oxidase Subunit VIIIA (*COX8A*)	NM_174024	Oxidative Phosphorylation	Fwd: TTTGACTTCGCGACCTTGG	60	This study
			Rev: TTACGGCACGGAGTAGACTG		
Solute Carrier Family 25 Member 39 (*SLC25A39*)	NM_001075415	Mitochondrial substrate/solute carrier	Fwd: AGCTAATGCCTCCCTCCAGA	54	This study
			Rev: GGCACTTCCATTTGGCGTAG		
Pyruvate Kinase M2 (*PKM2*)	NM_001205727	Glycolysis, Gluconeogenesis	Fwd: TGTCACCCATTACCAGCGAC	130	This study
			Rev: TATCTGGCCACCTGATGTGC		
SUZ12 polycomb repressive complex 2 subunit (*SUZ12*)	NM_001205587	Endogenous control	Fwd: CATCCAAAAGGTGCTAGGATAGATG	160	[62]
			Rev: TGGGCCTGCACACAAGAATG		

All samples were analyzed in triplicate during qPCR using the StepOnePlus Real-Time PCR System (Applied Biosystems). Each reaction contained 10 μL Fast SYBR® Green Master Mix (Applied Biosystems), 1 μL forward primer (20 pmol/μL), 1 μL reverse primer (20 pmol/μL), 7 μL nuclease-free water, and 1 μL cDNA template (2.5 ng/μL). The amplification program consisted of preincubation at 95 °C for 20 s followed by 40 cycles of denaturation at 95 °C for 3 s and annealing/extension at 64 °C for 30 s. Melting curve analysis was performed to confirm single product amplification. The expression of three housekeeping genes (*RPS18* (Ribosomal Protein S18), *GAPDH* (Glyceraldehyde 3-phosphate dehydrogenase), *SUZ12* (SUZ12 polycomb repressive complex 2 subunit)) was tested in this study using NormFinder [39] and PROC MIXED procedure of SAS (v9.2; SAS Institute Inc., Cary, NC). We chose *SUZ12* because it was the most stably expressed between and within the two groups. For each sample, the expression of each targeted gene was presented as ΔCt, which was obtained by subtracting the average Ct (Cycle threshold) of the *SUZ12* housekeeping gene from the average Ct of the target gene. A low ∆Ct indicates higher expression and vice versa. An unpaired t-test in SAS (SAS Institute Inc.) was used to analyze whether a gene showed significant difference between RFI groups.

### DNA Extraction

Total DNA was extracted and used to quantify the relative mitochondrial genome copy numbers. The DNA was extracted from the rumen papillae (~80 mg) of 104 animals (35 L-RFI, 34 M-RFI, 35 H-RFI) using a bead beating, phenol-chloroform method as described by Li et al., 2012 [40]. Briefly, each sample was placed in a 2-mL microcentrifuge tube containing zirconium beads (0.3 g; 0.1 mm diameter) and washed twice with 1 mL of TN150 buffer (10 mM Tris–HCl [pH 8.0], 150 mM NaCl) by vortexing and centrifugation at 14,600 g for 5 min at 4 °C. The tissue was then resuspended in 1 mL of TN150 and the Mini-BeadBeater-8 (BioSpec products, Bartlesville, OK) was used to mechanically disrupt cells at 5000 rpm for 3 min. After bead beating, samples were immediately placed on ice and washed two times by phenol and chloroform-isoamyl alcohol (24:1). The DNA was then precipitated at −20 °C overnight using cold 100 % ethanol and 3 M sodium acetate. After precipitation, DNA was washed two times with 70 % ethanol and dissolved in 30 μL nuclease-free water. The DNA concentration and quality was measured using Nanodrop (Thermo Scientific).

### qPCR determination of relative mitochondrial DNA copy number per cell

The relationship between RFI and the relative copy number of mitochondrial DNA (mtDNA) per cell of the rumen epithelium was performed using correlation analysis. The ratio of mtDNA to nuclear DNA (nDNA) was used to determine the relative copy number of mtDNA per cell. We selected the *ND1* (NADH dehydrogenase subunit 1) gene as the mtDNA target (GenBank accession number: NC_006853, Location: 3101–4056) and the single copy, *DDX3Y* (DEAD box polypeptide 3, Y-linked) gene as the nDNA target (GenBank accession number: AC_000187, Location: 3458510–3468728,). The *ND1* primer sequences used to amplify a 200 bp product were 5-GAACCACTACGACCCGCTACA-3 (forward), 5-GAGTTGGAAGCTCAGCCTGATC-3 (reverse) and the *DDX3Y* primer sequences used to amplify a 136 bp product were 5-ATCGTGGGCGGAATGAGTGT-3 (forward), 5-CTTGGTGGAAGCGGTTTTGA-3 (reverse) [41].

For qPCR, a standard curve was generated for both the mtDNA and nDNA. First, PCR was performed on pooled DNA to amplify mtDNA and nDNA separately. The PCR mix contained 50 ng/μL DNA along with 10X PCR buffer, 50 mM MgCl_2_, 10 mM dNTP, 20 pmol of each primer, Taq polymerase, and nuclease-free water to a final volume of 50 μL. The PCR program consisted of 1 cycle 94 °C for 5 min, 30 cycles of 94 °C for 30 s, 62 °C for 30 s, 72 °C for 30 s and 1 cycle of 72 °C for 7 min with hold at 4 °C. Each PCR product was then subjected to agarose gel electrophoresis in order to excise DNA bands for purification using the QIAquick Gel Extraction Kit (Qiagen). The concentration of the purified mtDNA and nDNA was measured by Nanodrop (Thermo Scientific) and an eight-point standard curve with 10 fold dilutions was created for each. DNA copy number of the standard curve points was calculated as described by Speicher and Johnson (2012) [42]. Expression of mtDNA and nDNA within 104 samples (35 L-RFI, 34 M-RFI, 35 H-RFI) was then analyzed by qPCR along with their respective standard curve using an annealing temperature of 62 °C. DNA copy number within samples was extrapolated from the standard curve and all samples and standard curves were analyzed in triplicate. A one-way ANOVA was performed in SAS (SAS Institute Inc.) to compare RFI groups. Also, a Pearson correlation analysis of relative mitochondrial DNA copy number per cell and RFI was performed in SAS (SAS Institute Inc.) and R [30].

## Results

### Identification of the rumen epithelial transcriptome

**Fig. 1:**
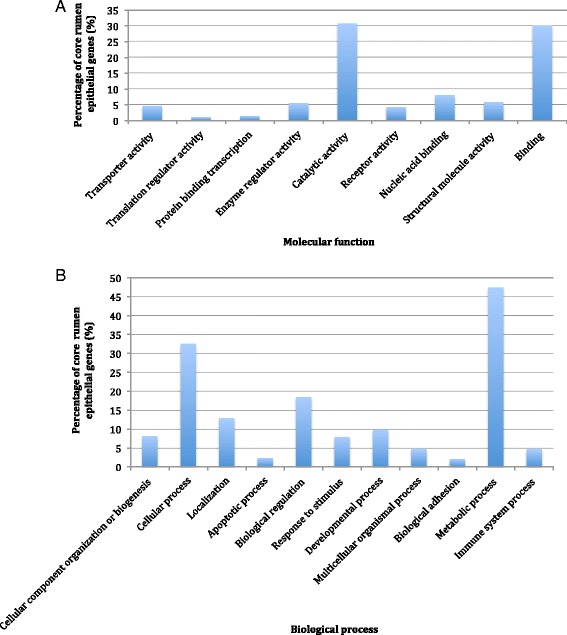
Biological functions of the core rumen epithelial genes as identified by PANTHER. The percentage of 9700 core rumen epithelial genes involved in various (**a**) Molecular functions and (**b**) Biological processes

On average there were 42 million high-quality, paired reads generated per sample (mean ± SD = 41,991,418 ± 6,835,817) from RNA-seq and the overall read alignment rate to the bovine reference genome was 85 ± 3.72 %. The total number of genes expressed (genes with at least one read in at least one sample) within the rumen epithelium was 22,338 with an average of 14,677 ± 1062 genes per L-RFI sample and 16,239 ± 1326 per H-RFI sample. There were 11,284 core genes (genes with at least one read in every sample) among samples and of these core genes, 9700 can be annotated by PANTHER. The top molecular functions for the core genes were catalytic activity and binding, which included 30.7 and 30.1 % of the core genes, respectively. In the biological processes category, 32.6 % of the core genes were involved in cellular process and 47.5 % of the genes were associated with metabolic process (Fig. 1).

### Comparison of transcriptome profiles between two RFI groups

**Fig. 2:**
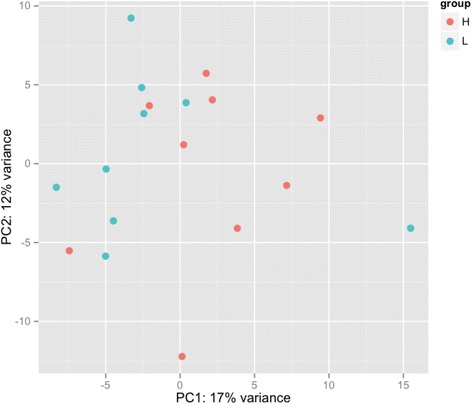
PCA plot of the rumen epithelial transcriptomes from cattle with L- and H- RFI. The L-RFI (Blue, *n* = 9) and H-RFI (Red, *n* = 9) samples are plotted along the first two principal components axis (PC1 and PC2)

**Fig. 3:**
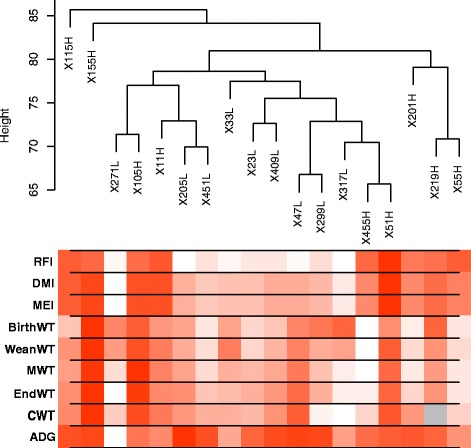
Hierarchical clustering dendrogram of rumen epithelial transcriptomes (9 L-RFI, 9 H-RFI) and trait heat map. The gradient from white to dark red in the trait heat map represents low to high levels of the trait while grey represents unavailable data. Traits examined were residual feed intake (RFI), dry matter intake (DMI), metabolizable energy intake (MEI), birth weight (BirthWT), weaning weight (WeanWT), metabolic mid-weight (MWT), end weight (EndWT), carcass weight (CWT), and average daily gain (ADG)

The PCA plot (Fig. 2) shows that L-RFI and H-RFI gene expression profiles seem to fall into distinct clusters, with those from L-RFI animals grouped together. However, there was a L-RFI outlier sample (271 L) that did not cluster with the rest of the samples (Fig. 2), which may possibly be related to the animal’s low ADG and EndWT (Fig. 3). Hierarchical clustering by WGCNA showed that all the L-RFI samples clustered together while the H-RFI samples did not (Fig. 3). When comparing animals with similar ADG and EndWT in the trait heat map, L-RFI individuals had lower RFI, DMI and MEI than H-RFI individuals. The means of the two groups for all traits (Additional file 2) were compared and there was no significant difference in BirthWT, WeanWT, MWT, EndWT, CWT, and ADG. However, the L-RFI group had significantly (*p* < 0.001) lower RFI, DMI and MEI by 3.82 kg/day, 3.86 kg/day and 4.68 Mcal/day, respectively.

### Differential gene expression in rumen epithelium between low- and high- RFI cattle

There were 122 genes DE in the rumen epithelial tissues between L- and H- RFI animals (Additional file 3). Of these DE genes, 85 were up-regulated and 37 down-regulated in the L-RFI group. DAVID annotation of the 122 DE genes found no significant KEGG pathways, however the biological term acetylation was significantly enriched (FDR < 0.05). A total of 30 DE genes were involved in the process of acetylation, 26 of which were up-regulated and 4 down-regulated in L-RFI animals (Table 2). These genes have functions in protein synthesis (i.e. *RPL10*, *RPS15*, *RPL36*), protein degradation (i.e. *PSMB6*, *UBC*/*UBA52*, *UBE2V1*), cytoskeletal organization (i.e. *DSTN*, *ACTB*, *TUBB5*, *TMSB10*), energetic substrate generation (i.e. *TECR*, *TPI1*), and stress response (i.e. *HSPB1*, *HSF1*).

**Table 2:** Rumen epithelial DE genes between L- and H- RFI steers significantly (FDR < 0.05) involved in acetylation

Ensembl ID	Gene symbol	Gene name	Fold change^a^	FDR adjusted p-value
ENSBTAG00000005654	*TMSB10*	Thymosin beta 10	1.44	1.28E-02
ENSBTAG00000012632	*TECR*	Trans-2,3-enoyl-CoA reductase	1.43	1.02E-05
ENSBTAG00000030974	*TUBA4A*	Tubulin, alpha 4a	1.43	5.63E-04
ENSBTAG00000007454	*RPL10*	Ribosomal protein L10	1.40	2.03E-04
ENSBTAG00000011969	*HSPB1*	Heat shock 27 kDa protein 1	1.34	1.28E-02
ENSBTAG00000013390	*PSMB6*	Proteasome (prosome, macropain) subunit, beta type, 6	1.32	1.41E-02
ENSBTAG00000016874	*DNAJB1*	DnaJ (Hsp40) homolog, subfamily B, member 1	1.29	1.28E-02
ENSBTAG00000019718	*RPS15*	Ribosomal protein S15	1.29	1.85E-03
ENSBTAG00000006969	*TUBB5*	Tubulin, beta	1.28	4.02E-02
ENSBTAG00000001794	*RPL36*	Ribosomal protein L36	1.28	2.49E-02
ENSBTAG00000020560	*CLPTM1*	Cleft lip and palate associated transmembrane protein 1	1.27	1.41E-02
ENSBTAG00000004379	*ETHE1*	Ethylmalonic encephalopathy 1	1.26	3.75E-02
ENSBTAG00000000411	*HGS*	Hepatocyte growth factor-regulated tyrosine kinase substrate	1.26	1.28E-02
ENSBTAG00000020751	*HSF1*	Heat shock transcription factor 1	1.26	2.84E-02
ENSBTAG00000021455	*CFL1*	Cofilin 1 (non-muscle)	1.26	1.41E-02
ENSBTAG00000014872	*CAPNS1*	Calpain, small subunit 1	1.26	1.36E-02
ENSBTAG00000016952	*PSMD5*	Proteasome (prosome, macropain) 26S subunit, non-ATPase, 5	1.26	4.07E-02
ENSBTAG00000026199	*ACTB*	Actin, beta	1.24	3.13E-02
ENSBTAG00000017246; ENSBTAG00000007737	*UBC; UBA52*	Ubiquitin C; Ubiquitin A-52 residue ribosomal protein fusion product 1	1.24	1.91E-02
ENSBTAG00000016024	*MYL9*	Myosin regulatory light polypeptide 9	1.24	6.77E-03
ENSBTAG00000019782	*TPI1*	Triosephosphate isomerase 1	1.24	3.56E-02
ENSBTAG00000006495	*GNB2*	Guanine nucleotide binding protein (G protein), beta polypeptide 2	1.24	4.89E-02
ENSBTAG00000027316	*UBE2V1*	Ubiquitin-conjugating enzyme E2 variant 1	1.23	4.02E-02
ENSBTAG00000015434	*DSTN*	Destrin (actin depolymerizing factor)	1.22	4.86E-02
ENSBTAG00000000215	*GNB1*	Guanine nucleotide binding protein (G protein), beta polypeptide 1	1.18	4.06E-02
ENSBTAG00000013362	*DNM2*	Dynamin 2	1.17	4.86E-02
ENSBTAG00000009780	*GTF2I*	General transcription factor II, i	0.85	4.86E-02
ENSBTAG00000009541	*SUCLG2*	Succinate-CoA ligase, GDP-forming, beta subunit	0.84	4.86E-02
ENSBTAG00000038488	*TMSB4*	Thymosin beta 4, X-linked	0.77	1.60E-03
ENSBTAG00000013982	*UACA*	Uveal autoantigen with coiled-coil domains and ankyrin repeats	0.72	2.11E-02

^a^Fold change is gene expression in L-RFI relative to H-RFI

Ingenuity analysis of the 85 differentially up-regulated genes in L-RFI samples showed that the top significantly enriched canonical pathway was remodeling of the epithelial adherens junctions through endocytosis (i.e. *DNM2*), degradation (i.e. *HGS*), and cytoskeletal organization (i.e. *ACTB*, *TUBB*, *TUBA4A*) (Table 3 and Additional file 3). The other enriched canonical pathways were involved in functions such as protein synthesis (eIF2 signaling), cytoskeletal dynamics, cell growth, proliferation, apoptosis, and migration (signaling by Rho family GTPases, RhoGDI signaling, ephrin B signaling) (Table 3). For the 37 DE genes that were down-regulated in L-RFI animals, there were no significant canonical pathways.

**Table 3:** Top Ingenuity canonical pathways significantly enriched (*p* < 0.05) for up- and down- regulated DE genes in L-RFI rumen epithelium

Ingenuity canonical pathways	FDR adjusted *p*-value	Genes
Up-regulated		
Remodeling of Epithelial Adherens Junctions	1.51E-03	*ACTB, TUBB5, DNM2, TUBA4A, HGS*
Signaling by Rho Family GTPases	2.42E-03	*RHOG, CFL1, ACTB, MYL12B, GNB1, MAPK1, GNB2*
RhoGDI Signaling	2.58E-03	*RHOG, CFL1, ACTB, MYL12B, GNB1, GNB2*
eIF2 Signaling	3.11E-03	*UBA52, RPL36, MAPK1, RPL18A, RPS15, RPL10*
Ephrin B Signaling	5.40E-03	*CFL1, GNB1, MAPK1, GNB2*

### qPCR validation of selected DE genes

To show the reliability of the differential gene expression analysis of DESeq2, we further selected five DE genes for validation using qPCR. As shown in Table 4, of these five genes, four (*TECR*, *COX8A*, *SLC25A39*, *PKM2*) had significantly greater expression (i.e. Lower ∆Ct, *p* < 0.05) in L-RFI while one (*TPI1*) tended to have significantly greater expression (*p* < 0.1). These results were in agreement with the differential expression analysis of DESeq2 in that the genes had greater expression in L-RFI rumen epithelium.

**Table 4:** Gene expression comparison between the rumen epithelium of L- and H- RFI animals by qPCR

Gene	L-RFI ∆Ct (*n* = 30)	H-RFI ∆Ct (*n* = 27)	*P*-value
*TPI1*	−3.23 ± 0.57	−2.99 ± 0.38	0.07
*TECR*	−2.36 ± 0.57	−2.00 ± 0.51	0.01*
*COX8A*	−7.85 ± 1.30	−6.57 ± 1.89	0.004*
*SLC25A39*	−3.67 ± 0.51	−0.24 ± 0.60	3.8E-30*
*PKM2*	−6.64 ± 0.58	−3.78 ± 0.53	4.4E-26*

Data are Mean ± SD

*p-value <0.05

### Identification of WGCNA gene co-expression modules correlated with RFI

**Fig. 4:**
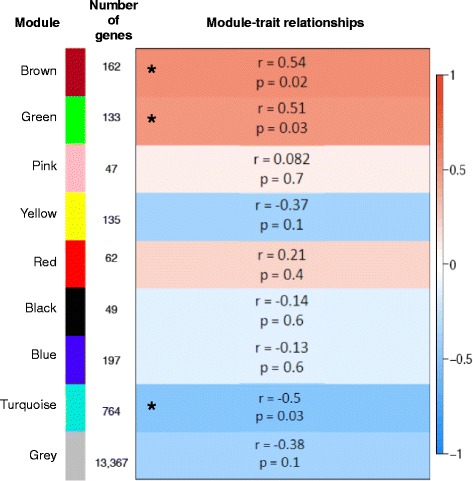
WGCNA identification of rumen epithelial gene modules correlated with the RFI trait. Module names and sizes are shown along with the module-trait relationships indicating the correlation coefficients and p-values. The strength of the correlation is colored by different intensities of red (positive correlation) and blue (negative correlation). Asterisks indicate modules that are significantly (*p* < 0.05) correlated with RFI

WGCNA was conducted to identify gene co-expression modules that are correlated with RFI. We identified a total of eight co-expressed gene modules with three of them significantly correlated with RFI (Fig. 4). The Brown (162 genes) and Green (133 genes) modules had a significant positive correlation with RFI (Brown module: *r* = 0.54, *p* = 0.02; Green module: *r* = 0.51, *p* = 0.03) while the Turquoise module (764 genes) had a significant negative correlation with RFI (*r* = −0.50, *p* = 0.03). Functional analysis of the genes enriched in the Green module did not result in any significantly enriched biological functions or pathways while there was only one significant biological term, which was muscle protein containing four genes in the Brown module. Many significant biological functions were identified for the turquoise module with IPA and DAVID analysis (Tables 5 and 6) and therefore this study focused on functional examination of the 764 genes enriched in this module, which had greater expression in the L-RFI epithelium.

**Table 5:** Biological functions and pathways significantly (FDR < 0.05) enriched in the Turquoise module (764 genes) using DAVID

DAVID functional category	Biological term	Number of genes	FDR adjusted *p*-value
SP_PIR_Keyword	Acetylation	195	8.22E-44
	Phosphoprotein	210	9.16E-19
	Ribonucleoprotein	51	3.63E-17
	Cytoplasm	138	1.17E-16
	Ribosomal protein	44	2.53E-16
	Protein biosynthesis	20	2.24E-06
	Isopeptide bond	23	5.54E-06
	Proteasome	15	8.76E-06
	Ubl conjugation	29	5.80E-05
	Nucleotide-binding	74	3.65E-04
	Wd repeat	22	8.04E-04
	Actin-binding	17	9.48E-04
	Transit peptide	35	4.94E-03
	Chaperone	17	6.61E-03
	Threonine protease	7	8.72E-03
	Methylation	17	1.67E-02
	Initiation factor	9	1.80E-02
	ATP-binding	50	2.43E-02
	Mitochondrion	47	3.18E-02
	Protein transport	24	3.47E-02
KEGG_Pathway	bta03010:Ribosome	36	3.05E-21
	bta03050:Proteasome	14	1.86E-05
	bta04520:Adherens junction	14	1.97E-03
	bta00190:Oxidative phosphorylation	19	5.94E-03
	bta04810:Regulation of actin cytoskeleton	22	2.65E-02
	bta03040:Spliceosome	16	3.95E-02

**Table 6:** Top biological functions and pathways significantly (*p* < 0.05) enriched in the Turquoise module with IPA

IPA category	Biological term	Number of genes	FDR adjusted *p*-value
Molecular and cellular function	Protein synthesis	89	6.06E-11
	Cellular growth and proliferation	232	6.06E-11
	Post-translational modification	51	2.80E-07
	Protein folding	17	7.92E-07
	Cell death and survival	211	1.03E-06
Canonical pathway	EIF2 signaling	51	5.85E-30
	Regulation of eIF4 and p70S6K signaling	35	3.28E-18
	mTOR signaling	39	3.60E-18
	Remodeling of epithelial adherens junctions	19	3.21E-11
	Epithelial adherens junction signaling	25	9.05E-10

### Functional analysis of gene module negatively associated with RFI

**Fig. 5:**
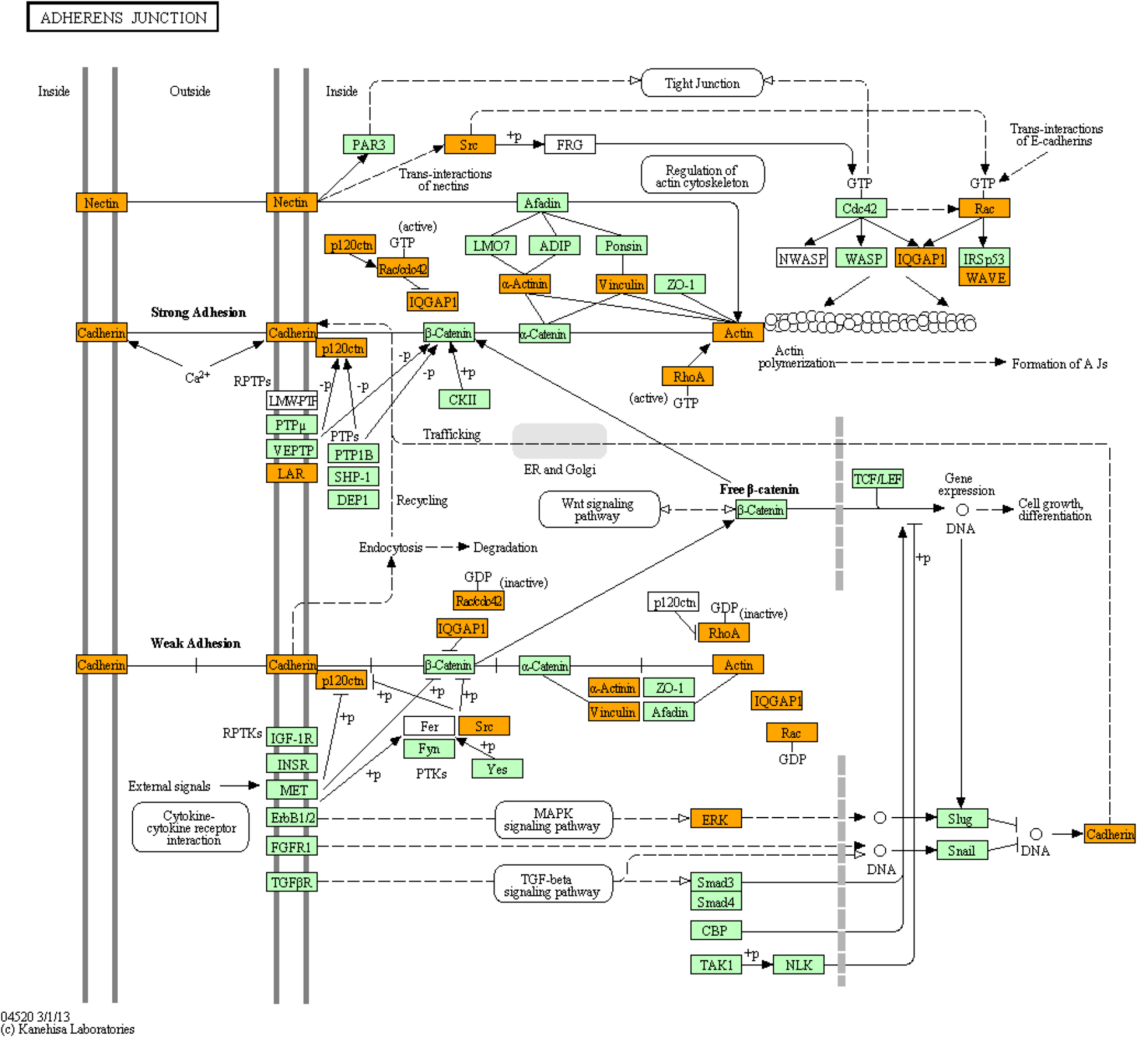
KEGG pathway for adherens junction. Genes within the turquoise module that have increased expression in the L-RFI rumen epithelium are shown in orange

When functional analysis was performed using DAVID, 20 biological functions and 6 KEGG pathways were significantly enriched for genes in the turquoise module (Table 5). The most significant biological functions were acetylation (195 genes) and phosphoprotein (210 genes), which are involved in post-translational modification. Many of the other significant biological functions identified were also involved in protein related processes such as protein synthesis (i.e. ribosomal protein (44 genes), protein biosynthesis (20 genes)), degradation (i.e. proteasome (15 genes), threonine protease (7 genes)), folding (i.e. chaperone (17 genes)), and transport (i.e. transit peptide (35 genes), protein transport (24 genes)). In addition, biological terms associated with energy catabolism (i.e. ATP-binding (50 genes)) and anabolism (i.e. mitochondrion (47 genes)) were also identified. KEGG analysis of the 195 genes within the acetylation category indicated significant involvement of the acetylation related genes in ribosome and proteasome pathways. These pathways were also significantly enriched in the turquoise module along with oxidative phosphorylation, adherens junction, regulation of actin cytoskeleton, and spliceosome (Table 5). In the adherens junction pathway, there were 14 genes from the turquoise module that may regulate both the strong and weak adhesion between cells (Fig. 5).

Further functional analysis using IPA showed that the top five most significant molecular and cellular functions for the genes in the turquoise module were protein synthesis, cellular growth and proliferation, post-translational modification, protein folding and cell death and survival (Table 6). Furthermore, the top canonical pathways were eIF2 signaling, regulation of eIF4 and p70S6K signaling, mTOR signaling and epithelial adherens junctions remodeling and signaling (Table 6).

Additionally, many genes from the turquoise module are involved in energy generating pathways such as glycolysis, tricarboxylic acid (TCA) cycle, and oxidative phosphorylation (Table 7). We also found three DE genes involved in glycolysis and three associated with oxidative phosphorylation, which were up-regulated in the L-RFI epithelium (Table 7). The DE genes involved in glycolysis were *TPI1* (Fold Change (FC; L-RFI/H-RFI) = 1.24), *HK1* (FC = 1.23), and *PKM2* (FC = 1.30) while the DE genes involved in oxidative phosphorylation were *COX8A* (FC = 1.28), *ATP6AP1* (FC = 1.39), and *ATP6V0D1* (FC = 1.26) (Table 7 and Additional file 3).

**Table 7:** Genes involved in major energy generating pathways with increased expression in the L-RFI rumen epithelium

Energy generating pathway	Gene symbol	Gene name	DE gene (D) and/or within turquoise module (T)
Glycolysis	*GPI*	Glucose-6-phosphate isomerase	T
	*PFKL*	Phosphofructokinase, liver	T
	*ALDOA*	Aldolase A, fructose-bisphosphate	T
	*GAPDH*	Glyceraldehyde-3-phosphate dehydrogenase	T
	*TPI1*	Triosephosphate isomerase 1	D, T
	*HK1*	Hexokinase 1	D
	*PKM2*	Pyruvate kinase M2	D
Tricarboxylic acid cycle	*CS*	Citrate synthase	T
	*SUCLG1*	Succinate-CoA ligase, alpha subunit	T
	*ACO2*	Aconitase 2, mitochondrial	T
	*SDHB*	Succinate dehydrogenase complex, subunit B	T
	*DLAT*	Dihydrolipoamide S-acetyltransferase	T
	*ACLY*	ATP citrate lyase	T
Oxidative phosphorylation	*ATP5D*	ATP synthase, H+ transporting, mitochondrial F1 complex, delta subunit	T
	*COX7A2L*	Cytochrome c oxidase subunit VIIa polypeptide 2 like	T
	*ATP5J2*	ATP synthase, H+ transporting, mitochondrial Fo complex, subunit F2	T
	*NDUFA10*	NADH dehydrogenase (ubiquinone) 1 alpha subcomplex, 10, 42 kDa	T
	*COX8A*	Cytochrome c oxidase subunit VIIIA (ubiquitous)	D, T
	*ATP6V1F*	ATPase, H+ transporting, lysosomal 14 kDa, V1 subunit F	T
	*PPA1*	Pyrophosphatase (inorganic) 1	T
	*UQCRQ*	Cytochrome b-c1 complex subunit 8	T
	*NDUFB10*	NADH dehydrogenase (ubiquinone) 1 beta subcomplex, 10, 22 kDa	T
	*NDUFS8*	NADH dehydrogenase (ubiquinone) Fe-S protein 8, 23 kDa (NADH-coenzyme Q reductase)	T
	*ATP6AP1*	ATPase, H+ transporting, lysosomal accessory protein 1	D, T
	*ATP6V0D1*	ATPase, H+ transporting, lysosomal 38 kDa, V0 subunit D1	D, T
	*ATP6V0E1*	ATPase, H+ transporting, lysosomal 9 kDa, V0 subunit E1	T
	*COX10*	Cytochrome c oxidase assembly protein	T
	*COX4I1*	Cytochrome c oxidase subunit IV isoform 1	T
	*UQCRC1*	Ubiquinol-cytochrome c reductase core protein I	T
	*ATP6V0A1*	ATPase, H+ transporting, lysosomal V0 subunit A1	T
	*NDUFV1*	NADH dehydrogenase (ubiquinone) flavoprotein 1, 51 kDa	T
	*PPA1*	Pyrophosphatase (inorganic) 1	T
	*SDHB*	Succinate dehydrogenase complex, subunit B, iron sulfur (Ip)	T

### Relative mtDNA copy number and RFI

**Fig. 6:**
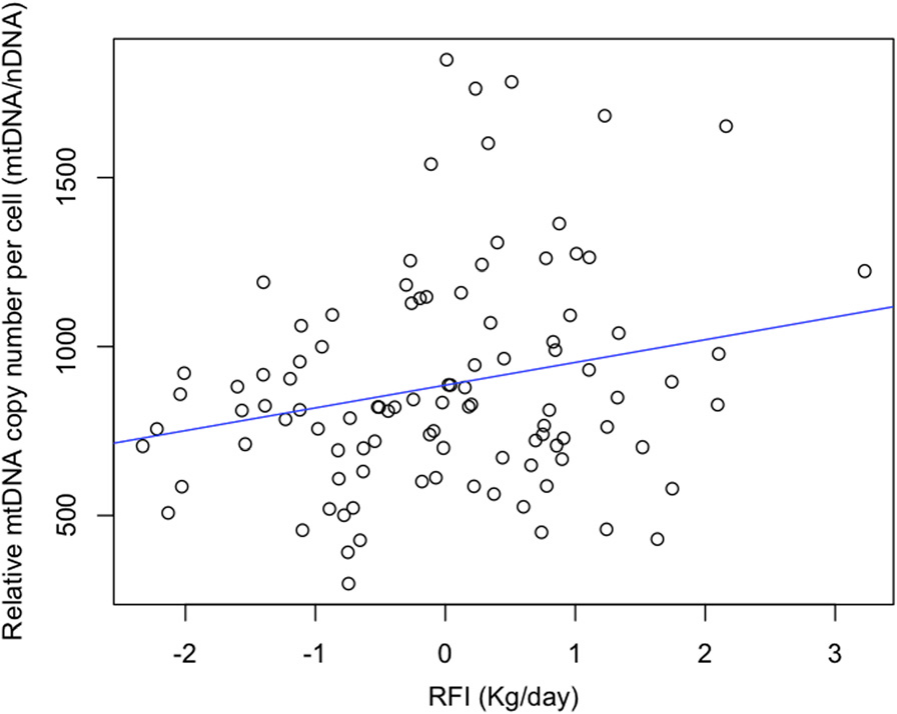
Correlation scatterplot of RFI and relative mtDNA copy number per cell of rumen epithelium (mtDNA/nDNA). Correlation coefficient (r) = 0.21, *p*-value = 0.03

The transcriptome analysis results showed up-regulation of genes in the L-RFI epithelium involved in mitochondrial functions such as oxidative phosphorylation. Therefore, we speculated that L-RFI animals would have more relative mtDNA copy numbers (mtDNA/nDNA) in the rumen epithelium than H-RFI animals. To test this hypothesis, we investigated the relationship between RFI and relative mtDNA copy number using a larger population including M-RFI (*n* = 34) animals along with L-RFI (*n* = 35) and H-RFI (*n* = 35) animals. The correlation analysis showed a significant positive relationship (*r* = 0.21, *p* = 0.03) between RFI and the relative mtDNA copy number per cell in the rumen epithelium (Fig. 6). The epithelium of L-RFI animals had 741 ± 35 (mean ± SD) relative mtDNA copy numbers per cell while the epithelium of M- and H- RFI animals had 1104 ± 89 and 1000 ± 80, respectively. There was a significantly greater copy number of mtDNA in the rumen epithelium of M- and H- RFI animals compared to L-RFI animals (*p* < 0.01). However, no significant difference in mtDNA copy number was observed between M- and H- RFI animals (*p* > 0.05).

## Discussion

Improving cattle feed efficiency could greatly reduce feed expense and increase profit for producers. Our study of steers divergent in feed efficiency as evaluated by RFI showed that L-RFI individuals are more feed efficient compared to H-RFI individuals because they were able to consume 3.82 kg less dry matter per day for similar growth, body weight, and carcass weight. These results coincide with those of others, which found that selection of L-RFI steers with 3.77 kg per day lower feed intake than H-RFI steers may lead to savings of $45.60 per head over a 120-day finishing period and there was no correlation of RFI with growth, body weight and carcass weight [4]. The difference in feed consumption between L- and H- RFI steers for similar production illustrates that differences may exist in the efficiency of feed conversion into energetic substrates, absorption, energy production, and in the distribution of energy for growth, maintenance, and activity requirements. Ruminants utilize the substrates produced by the rumen microbes for their growth. Studies have shown that the rumen microbial community differs between L- and H- RFI steers and may impact feed digestion and fermentation [43]. Although, the rumen microbial community has been associated with divergence in RFI, it is unknown whether there are differences in the rumen tissue that contribute to variation in RFI.

Our study found that 47.5 % of the core genes among the rumen epithelial tissues of beef steers are involved in metabolic processes. Indeed, the rumen epithelium was known to be highly metabolic because it contains a high density of mitochondria in the stratum basale layer [44] and metabolizes a large proportion of absorbed SCFAs [45]. Through hierarchical clustering, we found that the whole transcriptome profiles of the rumen epithelial tissues of H-RFI (inefficient) steers did not cluster together while all L-RFI (efficient) steers did. This indicates that the rumen epithelial transcriptome of H-RFI animals are more variable and there are similarities among the gene expression profiles of L-RFI animals that may be associated with higher feed efficiency.

We speculated that there was greater expression of genes involved in absorption of SCFAs from the rumen of L-RFI animals. However, we did not find evidence of differences in the expression of known or candidate SCFA transporters belonging to solute carrier (*SLC*) gene families such as *SLC4A*, *SLC16A* (monocarboxylate transporters), *SLC21A*, *SLC22A*, or *SLC26A* [19] between the L- and H- RFI animals in this study (data not shown). Although we did not observe differences in the expression of genes encoding SCFA transporters, our results do show evidence of differences in the expression of genes that are associated with paracellular permeability, which may impact simple diffusion of SCFAs and other nutrients. For example, there were 14 genes up-regulated in the L-RFI epithelium involved in the adherens junction pathway, which is important in modulating cell-cell adhesion [46, 47]. These genes encoded the plasma membrane cell adhesion molecules (*CAM*s), E-cadherin and nectin, as well as intracellular regulators of these molecules such as *p120ctn*, *v-Src*, *IQGAP1*, and *ACTB*. CAMs require attachment to the actin cytoskeleton in order to mediate cell adhesion [48]. Additionally, the E-cadherin CAM requires interaction with a key regulator, p120ctn, in order to promote strong adhesion between cells [49]. In our study, the L-RFI epithelium had increased expression of the *p120ctn* regulator, however, there was also increased expression of negative regulators such as *v-Src* and *IQGAP1* that promote weak adhesion. Furthermore, we found several DE genes that were up-regulated in the L-RFI epithelium encoding Dynamin-2 (*DNM2*), Hepatocyte growth factor-regulated tyrosine kinase substrate (*HGS*), and cytoskeletal components (Beta-actin (*ACTB*), Tubulin beta-5 (*TUBB5*), and Tubulin alpha-4a (*TUBA4A*)) that are involved in remodeling of epithelial adherens junctions. This suggests there is increased regulation of intercellular adhesion strength in the L-RFI epithelium. We also found that regulation of the actin cytoskeleton was a pathway that was up-regulated in the L-RFI epithelium. The regulation of intercellular adhesion strength and the actin cytoskeleton are vital to many processes such as cell migration, tissue remodeling, and maintenance of tissue integrity [50]. Indeed, we found up-regulated DE genes (*RHOG*, *CFL1, ACTB*, *MYL12B*, *GNB1*, *GNB2*, *MAPK1*) in the L-RFI epithelium involved in several important signaling pathways regulating cell migration including signaling by Rho family GTPases, RhoGDI, and Ephrin B that mediate their effects on cell migration though modulation of intercellular adhesion and regulation of the actin cytoskeleton [51–53]. Overall, the increased expression of genes related to modulation of intercellular adhesion strength, cytoskeletal organization, and cell migration signaling pathways suggest there is greater cell mobility and dynamic remodeling within the L-RFI epithelium, which may create gaps between cells resulting in greater paracellular permeability for the absorption of nutrients. Therefore, the L-RFI epithelium may potentially have increased paracellular permeability to maximize nutrient absorption in order to compensate for lower feed intake/substrate availability. Future studies are needed to examine the intercellular spaces, cell migration, and nutrient absorption in the rumen epithelium to determine whether they are truly different between steers divergent in RFI.

In addition to greater cell mobility and dynamic remodeling, further evidence from our transcriptome analyses results support that the rumen epithelium of L-RFI animals may have greater tissue morphogenesis compared to H-RFI animals. Tissue morphogenesis is a process that involves changes to the number, shape, size, position, and gene expression of cells in order to develop and maintain a tissue [54]. For instance, we found up-regulation of functions such as cellular growth, proliferation, and death in the L-RFI epithelium, which may indicate increased cell turnover. There was also up-regulation of protein synthesis, degradation, folding, transport, and post-translational modification processes that suggest increased protein turnover. Future studies to measure rumen papillae density/surface area or rumen volume are necessary to determine whether the L-RFI epithelium has increased tissue morphogenesis and permeability due to a larger surface area to volume ratio. Greater tissue morphogenesis suggests that the L-RFI epithelium may have greater cell growth and proliferation. This may be due to increased paracellular permeability causing the epithelial cells to be exposed to nutrients at a faster rate.

It is speculated that the difference in the expression of genes involved in tissue morphogenesis could be also associated with the variation in nutrient availability and flux. L-RFI animals had lower feed intake, suggesting that these animals may have lower microbial products available for the animals. However, it has been reported that L-RFI animals tended to have a greater concentration of total SCFAs (*p* = 0.059) in the rumen fluid with significantly greater concentrations of butyrate (*p* < 0.001) and valerate (*p* = 0.006) [43]. High concentrations of nutrients are associated with changes in the rumen epithelium such as extensive sloughing of surface epithelial cells, and increased cell migration and proliferation [55, 56]. Although the concentration of ruminal SCFAs was not measured for this study, the lack of difference in the expression of genes associated with SCFA oxidation and ketogenesis between L- and H- RFI suggest that the groups of animals may have similar SCFA levels in the rumen, although low RFI animals eat less. On the other hand, in L-RFI animals, nutrient flux may have been increased to maximize absorption because they have less substrate availability by consuming less feed. Regardless, future studies are needed to measure rate of nutrient absorption as well as the concentration of nutrients in the rumen content and epithelium to define their roles in affecting rumen tissue gene expression and in relation to RFI.

While there was no difference in the expression of SCFA metabolism genes, we found up-regulation of genes in the L-RFI epithelium involved in energy generating pathways such as glycolysis, TCA cycle, and oxidative phosphorylation. The majority of cellular energy, in the form of ATP, is generated through oxidative phosphorylation within the mitochondrial inner membrane [57]. Our results on increased expression of genes encoding mitochondrial proteins involved in oxidative phosphorylation in the L-RFI epithelium suggest that efficient steers may have increased energy production in the rumen epithelium. This may also indicate higher mitochondrial activity and/or greater mitochondrial genome copy numbers in the rumen epithelium of L-RFI steers compared to H-RFI steers. RFI was positively correlated (*r* = 0.21, *p* = 0.03), although very weakly, to the mitochondrial genome copy number per cell of the epithelium. Although there were fewer mitochondrial genomes in L-RFI animals, we observed increased expression of mitochondrial genes such as oxidative phosphorylation genes. This suggests that there is greater transcription from less genomes in the mitochondria of L-RFI animals. While we could not directly measure mitochondrial activity in our study, others have reported a more rapid rate of oxidative phosphorylation in the mitochondria of skeletal muscle and the liver in L-RFI beef cattle [58, 59]. We speculate that the mitochondria in the L-RFI rumen may also have the same capability, which needs to be further studied. Overall, increased expression of glycolytic and oxidative phosphorylation genes in the L-RFI epithelium suggests increased energy production that provides a resource for the increased tissue morphogenesis occurring to maintain barrier integrity in the L-RFI epithelium, which is an energetically expensive process. Although expensive, we speculate the L-RFI animals have enough energy for other processes throughout the body although they eat less. Future studies on examination of epithelial metabolism and whole body energy balance are needed to support our speculation.

Additionally, our study showed significant enrichment of genes in the L-RFI epithelium associated with acetylation, which is a post-translational modification process. These genes were involved with various processes such as protein synthesis, protein degradation, cytoskeletal organization, metabolism of energetic substrates, and stress response. This suggests that acetylation modulates genes belonging to a wide range of cellular processes. Acetylation is known to be a metabolically sensitive protein modification process [60]. It is known that approximately 35 % of all mitochondrial proteins are acetylated and the majority of these acetylated proteins are involved in energy metabolism [61]. The expression level of genes encoding proteins that have the potential to become acetylated could be an indicator of the level of energy metabolism occurring within animals. Our results on the increased expression of mitochondrial and metabolic genes suggest greater activity of the enzymes and more energy flow in low RFI animals.

## Conclusions

The RNA-seq based transcriptome analysis showed increased expression of genes in the L-RFI epithelium involved in modulation of adherens junctions, cytoskeletal organization, and cell migration signaling pathways that may cause large intercellular gaps and result in greater paracellular absorption of nutrients. Also, the L-RFI epithelium showed increased tissue morphogenesis as characterized by increased expression of genes involved in cell and protein turnover, modulation of intercellular adhesion strength, and cell migration, which may be due to increased absorption rate or a larger surface area to volume ratio. There was no difference in SCFA metabolism; however, there was increased expression of genes associated with glycolysis and oxidative phosphorylation in the L-RFI epithelium suggesting greater energy production. The increase in energy production provides a resource for the dynamic processes involved in maintaining tissue barrier integrity. A limitation of this study was that the epithelial tissue used for RNA-seq was obtained from only one location within the rumen. Pooling epithelial samples from multiple locations in the rumen is recommended to provide a better representation of the host transcriptome. Also, the epithelial tissue was acquired from animals only after slaughter. The transcriptome within animals may vary throughout their life and therefore it would be optimal to obtain epithelial samples from live animals through biopsy at various time points throughout the day and at different ages of the animal. Another limitation was that rumen size/volume and tissue morphology (i.e. rumen papillation parameters) was not examined, and the concentration and absorption of ruminal SCFAs could not be measured. Future work is needed to determine whether feed efficient steers truly have increased simple diffusion of SCFAs through the rumen wall, increased tissue morphogenesis, and increased energy production for the epithelium and for whole body energy use.

Regardless, this is one of the first efforts to study rumen epithelium in terms of its function and role in feed efficiency and our results suggest that the rumen epithelium may contribute to variation in RFI through differences in the expression of genes that affect the paracellular transport of nutrients, tissue morphology, and mitochondrial function. This provides a fundamental understanding on how host gene expression profiles are associated with RFI, which could potentially lead to identification of biomarkers for selection and improvement of cattle feed efficiency.

## Additional files

Additional file 1: RFI values of animals selected for transcriptome analysis. (DOCX 36 kb) Additional file 2: Phenotypic measures of the animals used for transcriptome profiling analysis. (DOCX 52 kb) Additional file 3: List of 122 genes DE in the rumen epithelial tissues between L- and H- RFI animals. (DOCX 145 kb)
