# Supplementary material for: Transcriptome profiling of the rumen epithelium of beef cattle differing in residual feed intake
Source: BMC Genomics. 2016 Aug 9;17:592. doi: 10.1186/s12864-016-2935-4 (PMC4979190; doi:10.1186/s12864-016-2935-4)
Supplement: Additional file 1: — RFI values of animals selected for transcriptome analysis. (DOCX 36 kb) [file 12864_2016_2935_MOESM1_ESM.docx]

| Animal ID | RFI |
| --- | --- |
| 317 | -2.33 |
| 205 | -2.22 |
| 47 | -2.13 |
| 271 | -2.04 |
| 33 | -2.01 |
| 23 | -1.60 |
| 409 | -1.57 |
| 451 | -1.54 |
| 299 | -1.40 |
| 201 | 1.32 |
| 219 | 1.52 |
| 105 | 1.63 |
| 155 | 1.74 |
| 455 | 1.75 |
| 115 | 2.10 |
| 55 | 2.10 |
| 11 | 2.16 |
| 51 | 3.23 |
