# Supplementary material for: Transcriptome profiling of the rumen epithelium of beef cattle differing in residual feed intake
Source: BMC Genomics. 2016 Aug 9;17:592. doi: 10.1186/s12864-016-2935-4 (PMC4979190; doi:10.1186/s12864-016-2935-4)
Supplement: Additional file 2: — Phenotypic measures of the animals used for transcriptome profiling analysis. (DOCX 52 kb) [file 12864_2016_2935_MOESM2_ESM.docx]

| Trait | L-RFI group (efficient; n = 9) | H-RFI group (inefficient; n = 9) | p-value |
| --- | --- | --- | --- |
| RFI (kg/day) | -1.87 ± 0.34 | 1.95 ± 0.56 | <0.001 |
| DMI (kg/day) | 8.52 ± 1.14 | 12.38 ± 1.25 | <0.001 |
| MEI (Mcal/day) | 10.34 ± 1.38 | 15.02 ± 1.52 | <0.001 |
| BirthWT (kg) | 43.10 ± 5.10 | 43.89 ± 8.43 | 0.973 |
| WeanWT (kg) | 543.20 ± 50.19 | 585.33 ± 109.60 | 0.398 |
| MWT (kg) | 99.71 ± 4.52 | 104.2 ± 9.79 | 0.226 |
| EndWT (kg) | 543.73 ± 41.78 | 575.30 ± 63.47 | 0.231 |
| CWT (kg) | 298.98 ± 23.54 | 322.24 ± 28.05 | 0.083 |
| ADG (kg/day) | 1.73 ± 0.41 | 1.78 ± 0.15 | 0.696 |

Data are Mean ± SD
