# Supplementary material for: Transcriptome profiling of the rumen epithelium of beef cattle differing in residual feed intake
Source: BMC Genomics. 2016 Aug 9;17:592. doi: 10.1186/s12864-016-2935-4 (PMC4979190; doi:10.1186/s12864-016-2935-4)
Supplement: Additional file 3: — List of 122 genes DE in the rumen epithelial tissues between L- and H- RFI animals. (DOCX 145 kb) [file 12864_2016_2935_MOESM3_ESM.docx]

| **Ensembl ID** | **Gene symbol** | **Gene name** | **Fold change*** | **FDR adjusted p-value** |
| --- | --- | --- | --- | --- |
| ENSBTAG00000033423 | *EFNA3* | Ephrin-A3 | 1.48 | 3.56E-02 |
| ENSBTAG00000007583 | *KRT14* | Keratin 14 | 1.47 | 3.91E-02 |
| ENSBTAG00000008331 | *TMEM54* | Transmembrane protein 54 | 1.47 | 1.28E-02 |
| ENSBTAG00000005654 | *TMSB10* | Thymosin beta 10 | 1.44 | 1.28E-02 |
| ENSBTAG00000015145 | *S100A11* | S100 calcium binding protein A11 (calgizzarin) | 1.43 | 4.08E-02 |
| ENSBTAG00000009047 | *YPEL3* | Yippee-like 3 (Drosophila) | 1.43 | 1.62E-02 |
| ENSBTAG00000012632 | *TECR* | Trans-2,3-enoyl-CoA reductase | 1.43 | 1.02E-05 |
| ENSBTAG00000030974 | *TUBA4A* | Tubulin, alpha 4a | 1.43 | 5.63E-04 |
| ENSBTAG00000002631 | *SCNN1A* | Sodium channel, nonvoltage-gated 1 alpha | 1.41 | 3.60E-02 |
| ENSBTAG00000046339 | *VASN* | Vasorin | 1.41 | 4.89E-02 |
| ENSBTAG00000001207 | *SERPINB8* | Serpin peptidase inhibitor, clade B (ovalbumin), member 8 | 1.41 | 4.00E-02 |
| ENSBTAG00000015313 | *CEACAM19* | Carcinoembryonic antigen-related cell adhesion molecule 19 | 1.40 | 1.41E-02 |
| ENSBTAG00000007454 | *RPL10* | Ribosomal protein L10 | 1.40 | 2.03E-04 |
| ENSBTAG00000015908 | *MBOAT7* | Membrane bound O-acyltransferase domain containing 7 | 1.39 | 1.91E-02 |
| ENSBTAG00000012117 | *ATP6AP1* | ATPase, H+ transporting, lysosomal accessory protein 1 | 1.39 | 2.03E-04 |
| ENSBTAG00000000347 | *RHOG* | Ras homolog gene family, member G (rho G) | 1.39 | 1.28E-02 |
| ENSBTAG00000025161 | *AGPAT2* | 1-acylglycerol-3-phosphate O-acyltransferase 2 (lysophosphatidic acid acyltransferase, beta) | 1.38 | 2.21E-03 |
| ENSBTAG00000018077 | *LYPD3* | LY6/PLAUR domain containing 3 | 1.38 | 9.89E-04 |
| ENSBTAG00000001350 |  | Uncharacterized protein | 1.37 | 1.71E-03 |
| ENSBTAG00000018506 |  | Uncharacterized protein | 1.37 | 3.56E-02 |
| ENSBTAG00000000585 | *LY6G6C* | Lymphocyte antigen 6 complex, locus G6C | 1.37 | 1.41E-02 |
| ENSBTAG00000046100 |  | Uncharacterized protein | 1.37 | 2.84E-02 |
| ENSBTAG00000015920 | *TMEM147* | Transmembrane protein 147 | 1.35 | 1.28E-02 |
| ENSBTAG00000012046 | *JUNB* | Jun B proto-oncogene | 1.35 | 4.08E-02 |
| ENSBTAG00000025277 | *ABHD17A* | Family with sequence similarity 108, member A1 | 1.35 | 2.11E-02 |
| ENSBTAG00000009580 | *SH3BGRL3* | SH3 domain binding glutamic acid-rich protein like 3 | 1.34 | 1.71E-03 |
| ENSBTAG00000011969 | *HSPB1* | Heat shock 27kDa protein 1 | 1.34 | 1.28E-02 |
| ENSBTAG00000010626 | *SH3GLB2* | SH3-domain GRB2-like endophilin B2 | 1.33 | 2.44E-02 |
| ENSBTAG00000006241 | *MAN2B1* | Mannosidase, alpha, class 2B, member 1 | 1.33 | 1.28E-02 |
| ENSBTAG00000004875 | *NAT15* | N-acetyltransferase 15 (GCN5-related) | 1.32 | 4.86E-02 |
| ENSBTAG00000009183 | *SHISA5* | Shisa homolog 5 (Xenopus laevis) | 1.32 | 2.49E-02 |
| ENSBTAG00000013390 | *PSMB6* | Proteasome (prosome, macropain) subunit, beta type, 6 | 1.32 | 1.41E-02 |
| ENSBTAG00000001601 | *PKM2* | Pyruvate kinase, muscle | 1.30 | 1.28E-02 |
| ENSBTAG00000012726 | *PSMB5* | Proteasome (prosome, macropain) subunit, beta type, 5 | 1.30 | 3.48E-03 |
| ENSBTAG00000020998 | *RUVBL1* | RuvB-like 1 (E. coli) | 1.30 | 4.00E-02 |
| ENSBTAG00000030592 | *UBL5* | Ubiquitin-like 5 | 1.30 | 4.97E-02 |
| ENSBTAG00000010312 | *MAPK1* | Mitogen-activated protein kinase 1 | 1.30 | 1.28E-02 |
| ENSBTAG00000024091 | *MALL* | Mal, T-cell differentiation protein-like | 1.29 | 1.41E-02 |
| ENSBTAG00000016874 | *DNAJB1* | DnaJ (Hsp40) homolog, subfamily B, member 1 | 1.29 | 1.28E-02 |
| ENSBTAG00000008172 | *EGLN3* | Egl nine homolog 3 (C. elegans) | 1.29 | 1.60E-03 |
| ENSBTAG00000019718 | *RPS15* | Ribosomal protein S15 | 1.29 | 1.85E-03 |
| ENSBTAG00000019040 | *PLBD2* | Phospholipase B domain containing 2 | 1.29 | 3.01E-02 |
| ENSBTAG00000015406 | *ZNF750* | Zinc finger protein 750 | 1.29 | 1.32E-02 |
| ENSBTAG00000006969 | *TUBB5* | Tubulin, beta; similar to tubulin, beta 5 | 1.28 | 4.02E-02 |
| ENSBTAG00000004920 | *COX8A* | Cytochrome c oxidase subunit 8A (ubiquitous) | 1.28 | 4.61E-02 |
| ENSBTAG00000048098 | *PKP3* | Plakophilin 3 | 1.28 | 1.41E-02 |
| ENSBTAG00000001794 | *RPL36* | 60S ribosomal protein L36 | 1.28 | 2.49E-02 |
| ENSBTAG00000046350 | *PKP1* | Plakophilin 1 | 1.27 | 4.08E-02 |
| ENSBTAG00000016093 | *PLP2* | Proteolipid protein 2 (colonic epithelium-enriched) | 1.27 | 1.60E-03 |
| ENSBTAG00000020067 | *LLGL2* | Lethal giant larvae homolog 2 (Drosophila) | 1.27 | 3.56E-02 |
| ENSBTAG00000020560 | *CLPTM1* | Cleft lip and palate associated transmembrane protein 1 | 1.27 | 1.41E-02 |
| ENSBTAG00000004379 | *ETHE1* | Ethylmalonic encephalopathy 1 | 1.26 | 3.75E-02 |
| ENSBTAG00000019463 | *SLC25A39* | Solute carrier family 25, member 39 | 1.26 | 1.91E-02 |
| ENSBTAG00000000411 | *HGS* | Hepatocyte growth factor-regulated tyrosine kinase substrate | 1.26 | 1.28E-02 |
| ENSBTAG00000020751 | *HSF1* | Heat shock transcription factor 1 | 1.26 | 2.84E-02 |
| ENSBTAG00000021455 | *CFL1* | Cofilin 1 (non-muscle) | 1.26 | 1.41E-02 |
| ENSBTAG00000014872 | *CAPNS1* | Calpain, small subunit 1 | 1.26 | 1.36E-02 |
| ENSBTAG00000014553 | *ATP6V0D1* | ATPase, H+ transporting, lysosomal 38kDa, V0 subunit d1 | 1.26 | 4.86E-02 |
| ENSBTAG00000010663 | *ADAM15* | ADAM metallopeptidase domain 15 | 1.26 | 3.60E-02 |
| ENSBTAG00000016952 | *PSMD5* | Proteasome (prosome, macropain) 26S subunit, non-ATPase, 5 | 1.26 | 4.07E-02 |
| ENSBTAG00000014883 | *GABARAP* | GABA(A) receptor-associated protein | 1.25 | 3.54E-02 |
| ENSBTAG00000018914 | *RAB25* | RAB25, member RAS oncogene family | 1.25 | 3.38E-02 |
| ENSBTAG00000014265 | *SREBF2* | Sterol regulatory element binding transcription factor 2 | 1.25 | 1.28E-02 |
| ENSBTAG00000011904 | *HCFC1* | Host cell factor C1 (VP16-accessory protein) | 1.25 | 2.84E-02 |
| ENSBTAG00000027075 |  | Uncharacterized protein | 1.24 | 4.51E-02 |
| ENSBTAG00000015831 | *RPL18A* | Similar to ribosomal protein L18a; ribosomal protein L18a | 1.24 | 1.41E-02 |
| ENSBTAG00000026199 | *ACTB* | Actin, beta | 1.24 | 3.13E-02 |
| ENSBTAG00000017246;  ENSBTAG00000007737 | *UBC; UBA52* | Ubiquitin C; Ubiquitin A-52 residue ribosomal protein fusion product 1 | 1.24 | 1.91E-02 |
| ENSBTAG00000016024 | *MYL9* | Myosin regulatory light polypeptide 9 | 1.24 | 6.77E-03 |
| ENSBTAG00000019782 | *TPI1* | Triosephosphate isomerase 1 | 1.24 | 3.56E-02 |
| ENSBTAG00000006495 | *GNB2* | Guanine nucleotide binding protein (G protein), beta polypeptide 2 | 1.24 | 4.89E-02 |
| ENSBTAG00000023274 |  | Uncharacterized protein | 1.23 | 2.32E-02 |
| ENSBTAG00000006007 | *SH3GL1* | SH3-domain GRB2-like 1 | 1.23 | 6.36E-03 |
| ENSBTAG00000027316 | *UBE2V1* | Ubiquitin-conjugating enzyme E2 variant 1 | 1.23 | 4.02E-02 |
| ENSBTAG00000012380 | *HK1* | Hexokinase 1 | 1.23 | 3.38E-02 |
| ENSBTAG00000019851 | *PPP2R1A* | Protein phosphatase 2 (formerly 2A), regulatory subunit A, alpha isoform | 1.23 | 4.08E-02 |
| ENSBTAG00000031875 | *BANF1* | Barrier to autointegration factor 1 | 1.23 | 4.97E-02 |
| ENSBTAG00000015434 | *DSTN* | Destrin (actin depolymerizing factor) | 1.22 | 4.86E-02 |
| ENSBTAG00000019685 | *BAG6* | BCL2-associated athanogene 6 | 1.22 | 1.28E-02 |
| ENSBTAG00000009663 | *CSDA* | Cold shock domain protein A | 1.22 | 2.11E-02 |
| ENSBTAG00000002381 | *ZDHHC5* | Zinc finger, DHHC-type containing 5 | 1.21 | 2.44E-02 |
| ENSBTAG00000011484 | *ZDHHC3* | Zinc finger, DHHC-type containing 3 | 1.18 | 4.86E-02 |
| ENSBTAG00000000215 | *GNB1* | Guanine nucleotide binding protein (G protein), beta polypeptide 1 | 1.18 | 4.06E-02 |
| ENSBTAG00000013362 | *DNM2* | Dynamin 2 | 1.17 | 4.86E-02 |
| ENSBTAG00000011488 | *PRPF8* | PRP8 pre-mRNA processing factor 8 homolog (S. cerevisiae) | 1.16 | 2.50E-02 |
| ENSBTAG00000016080 | *VPS13D* | Vacuolar protein sorting 13 homolog D (S. cerevisiae) | 0.85 | 2.11E-02 |
| ENSBTAG00000009780 | *GTF2I* | General transcription factor II, i | 0.85 | 4.86E-02 |
| ENSBTAG00000009541 | *SUCLG2* | Succinate-CoA ligase, GDP-forming, beta subunit | 0.84 | 4.86E-02 |
| ENSBTAG00000021209 | *UBR5* | Ubiquitin protein ligase E3 component n-recognin 5 | 0.83 | 3.60E-02 |
| ENSBTAG00000008862 | *GOLGB1* | Golgin B1 | 0.82 | 2.84E-02 |
| ENSBTAG00000006940 | *USP48* | Ubiquitin specific peptidase 48 | 0.81 | 4.86E-02 |
| ENSBTAG00000009061 | *FAR1* | Fatty acyl CoA reductase 1 | 0.80 | 4.86E-02 |
| ENSBTAG00000020914 | *CPNE8* | Copine VIII | 0.79 | 4.06E-02 |
| ENSBTAG00000016038 | *GCC2* | GRIP and coiled-coil domain containing 2 | 0.79 | 2.84E-02 |
| ENSBTAG00000003697 | *TARDBP* | TAR DNA binding protein | 0.78 | 4.80E-02 |
| ENSBTAG00000020233 | *CCDC186* | Coiled-coil domain containing 186 | 0.78 | 4.86E-02 |
| ENSBTAG00000005443 | *MIER1* | Mesoderm induction early response 1 homolog (Xenopus laevis) | 0.78 | 2.49E-02 |
| ENSBTAG00000038488 | *TMSB4* | Thymosin beta 4, X-linked | 0.77 | 1.60E-03 |
| ENSBTAG00000027569 | *APBB2* | Amyloid beta (A4) precursor protein-binding, family B, member 2 | 0.77 | 4.00E-02 |
| ENSBTAG00000019500 | *CNIH1* | Cornichon homolog (Drosophila) | 0.76 | 2.59E-02 |
| ENSBTAG00000001485 | *PPIP5K2* | Diphosphoinositol pentakisphosphate kinase 2 | 0.76 | 1.41E-02 |
| ENSBTAG00000047537 | *CCAR1* | Cell division and apoptosis regulator 1 | 0.75 | 2.44E-02 |
| ENSBTAG00000015612 | *UTP6* | Small subunit processome component | 0.74 | 4.66E-02 |
| ENSBTAG00000047029 |  | Uncharacterized protein | 0.73 | 4.86E-02 |
| ENSBTAG00000014044 | *VEZT* | Vezatin, adherens junctions transmembrane protein | 0.73 | 4.18E-02 |
| ENSBTAG00000014099 | *YTHDC2* | YTH domain containing 2 | 0.73 | 2.84E-02 |
| ENSBTAG00000011187 | *FAM13A* | Family with sequence similarity 13, member A1 | 0.73 | 4.36E-02 |
| ENSBTAG00000014469 | *NBEAL1* | Neurobeachin-like 1 | 0.72 | 6.05E-03 |
| ENSBTAG00000003064 | *GCFC* | GC-rich sequence DNA-binding factor homolog | 0.72 | 4.86E-02 |
| ENSBTAG00000013982 | *UACA* | Uveal autoantigen with coiled-coil domains and ankyrin repeats | 0.72 | 2.11E-02 |
| ENSBTAG00000037440 |  | Uncharacterized protein | 0.71 | 4.18E-02 |
| ENSBTAG00000027728 | *NUDT12* | Nudix (nucleoside diphosphate linked moiety X)-type motif 12 | 0.71 | 1.28E-02 |
| ENSBTAG00000026233 |  | Uncharacterized protein | 0.70 | 2.11E-02 |
| ENSBTAG00000034580 | *TMSB4* | Thymosin beta-4 Hematopoietic system regulatory peptide | 0.70 | 1.41E-02 |
| ENSBTAG00000045772 |  | Uncharacterized protein | 0.69 | 1.37E-02 |
| ENSBTAG00000016932 | *SENP7* | SUMO1/sentrin specific peptidase 7 | 0.68 | 1.41E-02 |
| ENSBTAG00000009035 | *CENPE* | Centromere protein E, 312kDa | 0.68 | 2.84E-02 |
| ENSBTAG00000006255 | *MDM4* | Mdm4 p53 binding protein homolog (mouse) | 0.68 | 2.59E-02 |
| ENSBTAG00000042484 | *SNORD22* | Small nucleolar RNA SNORD22 | 0.67 | 4.00E-02 |
| ENSBTAG00000000782 | *KDR* | Kinase insert domain receptor (a type III receptor tyrosine kinase) | 0.65 | 2.84E-02 |
| ENSBTAG00000005445 | *SLC35D2* | Solute carrier family 35 (UDP-glucuronic acid/UDP-N-acetylgalactosamine dual transporter), member D1 | 0.65 | 5.63E-04 |
| ENSBTAG00000048293 | *U2* | U2 spliceosomal RNA | 0.64 | 2.84E-02 |

*Fold change is gene expression in L-RFI relative to H-RFI
